# *CDHR2* c.2233C > T Is Involved in Human Familial Ovarian Immature Teratoma With *BMP15* c.262C > T

**DOI:** 10.1155/humu/8441244

**Published:** 2026-06-19

**Authors:** Yuntao Hao, Xi Kang, Yakun Liu, Hongwei Fan, Yan Li, Shan Kang

**Affiliations:** ^1^ Department of Gynecology, The Fourth Hospital of Hebei Medical University, Shijiazhuang, China, hebmu.edu.cn; ^2^ Department of Obstetrics and Gynecology, Aerospace Center Hospital, Beijing, China, asch.net.cn; ^3^ Department of Surgery, The Fourth Hospital of Hebei Medical University, Shijiazhuang, China, hebmu.edu.cn; ^4^ Department of Molecular Biology, The Fourth Hospital of Hebei Medical University, Shijiazhuang, China, hebmu.edu.cn

**Keywords:** CDHR2, nonsense mutation, ovarian immature teratoma, whole-exome sequencing

## Abstract

We previously identified the germline mutation c.262C > T in *BMP15* as a genetic susceptibility factor of hereditary ovarian immature teratoma (OIT). However, not all female carriers in that family had OIT. In this study, we analyzed the genetic differences among these individuals, aiming to explore whether any genes may be copathogenic with *BMP15*. The subjects were recruited from two three‐generation pedigrees of hereditary OIT. Whole‐exome sequencing (WES) was performed, and the candidate variants were subsequently tested by Sanger sequencing. Moreover, bioinformatics was used to assess the mutation with MutationTaster and Combined Annotation Dependent Depletion (CADD), and pathogenicity was predicted according to the American College of Medical Genetics and Genomics (ACMG) guidelines. The effect of the mutation on CDHR2 expression was evaluated by RT–qPCR, Western blotting, and immunofluorescence with confocal microscopy. WES revealed that, unlike the two unaffected sisters, both affected second‐generation individuals harbored a germline mutation in *CDHR2* (c.2233C > T, p.R745X) in addition to the mutation in *BMP15* (c.262C > T, p.R86C). *CDHR2* c.2233C > T is a truncating mutation that was predicted to be deleterious by MutationTaster and CADD and classified as pathogenic according to the ACMG criteria. Our in vitro experiments revealed that mRNA and protein expression levels were significantly lower in cells with the 2233T allele than in those with the 2233C allele. Moreover, the localization of the mutant product in cells was significantly different from that of the wild‐type protein. The mutant group exhibited abnormal accumulation of small‐molecule metabolites, along with aberrant activation of the *HIPPO* signaling cascade. Our findings suggest that the *CDHR2* c.2233C > T variant may have a synergistic effect with *BMP15* in hereditary OIT or act as a modifier gene in OIT development.

## 1. Introduction

Ovarian immature teratoma (OIT) is a rare malignant tumor originating from female germ cells, with an incidence of approximately 3.4 × 10^−7^ [1]. OIT is a malignant tumor of the female reproductive system that exhibits complex tissue structures characteristic of all three embryonic germ layers; OITs typically occur in women younger than 30 years and impair patients′ reproductive health [2–4]. However, owing to its rarity and germ cell origin, the etiology of OIT is undefined. Next‐generation sequencing (including whole‐exome sequencing [WES] and whole‐genome sequencing [WGS]) and Sanger sequencing enable exploration of the associations between genetic variations and their interactions in some complex phenotypes, such as cardiovascular disease [5], Type 2 diabetes [6, 7], and familial peripheral neuroblastic tumors [8], and facilitate the dissection of their mechanisms, especially those involving oligogenic or polygenic inheritance and genetic modifiers. These methods were also beneficial for understanding the etiology of OIT in this study.

In our previous study, we identified a germline missense mutation in *BMP15* (c.262C > T, p.R86C) via WES and demonstrated that this mutation was involved in the pathogenesis of hereditary OIT [9]. However, only some individuals in the family harboring this mutation suffered from OIT. This finding led us to wonder whether other causative germline mutations synergistically interact with the *BMP15* mutation to cause hereditary OIT.

In this study, we used WES to analyze the differences in germline mutations between two sisters with OIT and their two healthy sisters carrying *BMP15* c.262C > T mutations in the same family. A mutation in *CDHR2* (c.2233C > T, rs200848414) was identified that may coparticipate with *BMP15* c.262C > T in the occurrence of hereditary OIT.

## 2. Materials and Methods

### 2.1. Study Subjects

**Figure 1:**
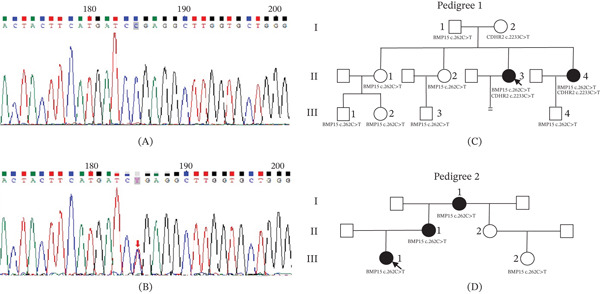
Mutations in familial OIT. (A) *CDHR2* c.2233C > T (p.R745X) was identified by Sanger sequencing. The mutation site is indicated by the red arrow. (B) Sanger sequencing results of individuals from families without *CDHR2* nonsense mutations. (C) The genealogy of the OIT lineage in our investigation is depicted using square symbols for males and round symbols for females. The affected individuals are marked in black, and the arrow denotes the proband. Genotypes of *BMP15* c.262C > T and *CDHR2* c.2233C > T are shown below each symbol.

The clinical data and blood samples used in this study were collected from two Chinese families with OIT (Pedigrees 1 and 2 in Figure 1). Pedigree 1 included two patients (II‐3 and II‐4) and eight healthy individuals (I‐1, I‐2, II‐1, II‐2, III‐1, III‐2, III‐3, and III‐4). Pedigree 2 comprised three patients (I‐1, II‐1, and III‐1) and two healthy individuals (II‐2 and III‐2), with the second‐generation individuals being maternal half‐sisters. All patients diagnosed with OIT met the diagnostic criteria for the disease. To investigate other potential genetic causes of familial OIT, we further analyzed the results of WES (presented in our previous study) of members II‐1, II‐2, II‐3, and II‐4 of Pedigree 1.

### 2.2. DNA Extraction, WES, and Genetic Analysis

Genomic DNA was isolated from venous blood as described in our previous study. The WES data used in this study were also based on the previous results of our team. Candidate variants were amplified by conventional PCR methods and validated by Sanger sequencing using specific primers by Shanghai Generay Biotech Co., Ltd. (https://www.generay.com.cn). Candidate pathogenic genes were defined as those with a rare exonic nonsynonymous variant (minor allele frequency [MAF] < 0.0001 in major databases, e.g., the *ExAC*, *GnomAD*, *GnomAD_exome*, and *TOPMed* population databases) that was present in all affected women. We subsequently searched the public databases for corresponding variants. Candidate mutations were also required to be predicted to be deleterious and linked to tumorigenesis. The pathogenicity of the detected variants was assessed by *MutationTaster* (https://www.Mutationtaster.org/) and Combined Annotation Dependent Depletion (*CADD*) (https://cadd.gs.Washington.edu). The variants are classified as benign if they have a CADD score below 20 and as damaging if they have a CADD score above 20 [10]. The genotypes of other family members in the pedigrees were also determined by Sanger sequencing. Finally, cosegregation of the candidate mutations with the family phenotype was analyzed using the precise cosegregation analysis method, which was proposed by the 2019 Expert Group on Deafness Variation Guidelines (cosegregation was defined as a *Z* value ≥ 0.6) [11].

### 2.3. Amplification and Sanger Sequencing

The genetic analyses above suggest that *CDHR2* mutations may contribute to OIT pathogenesis in this pedigree. The PCR primers for *CDHR2* amplification (Table S1) were designed using Premier 5.0 primer design software. The experimental procedures were conducted by Shanghai Lianghao Biotechnology Co., Ltd. The sequences were aligned with the genomic DNA sequence of *CDHR2*, and nucleotide variations were annotated according to their positions in the CDHR2 mRNA (NM_017675).

### 2.4. Construction of Plasmids Expressing CDHR2 and Its Mutant (MT)

The plasmids expressing CDHR2 and its MT, *CDHR2-zsGreen-3Flag* and *CDHR2(1-744)-zsGreen-3Flag*, were obtained from Jikai Company (Shanghai, China), while the GV657 vector was used as a negative control (NC). *CDHR2* variant cDNA was generated using site‐directed mutagenesis of the full‐length cDNA of the wild‐type (WT) human *CDHR2* gene, with the “C” base of the WT *CDHR2* gene replaced with a “T” base, leading to the formation of a premature stop codon. To characterize the subcellular localization of WT and MT CDHR2 and monitor truncated protein products, all cDNA constructs were modified to carry an N‐terminal 3Flag tag.

### 2.5. Cell Culture and Transfection

Human embryonic kidney 293T (HEK293T) cells were obtained as in our previous study. The cells were cultured in Dulbecco′s Modified Eagle′s Medium (Gibco, Thermo Fisher Scientific, Inc.) supplemented with 10% fetal bovine serum and 0.5% penicillin–streptomycin solution in a humidified atmosphere containing 5% CO_2_ at 37°C. HEK293T cells were transfected with Lipofectamine 2000 (Invitrogen) and the expression plasmids according to the manufacturer′s instructions. Transfection efficiency was initially evaluated by observing the green fluorescent protein under a fluorescence microscope after 24 h. Cells with a transfection efficiency greater than 80% were selected for subsequent experiments.

### 2.6. Reverse Transcription and Real‐Time Quantitative PCR (RT–qPCR)

HEK293T cells were harvested 24 h after transfection, and total RNA was extracted from the cells using TRIzol reagent (Generay Biotech [Shanghai], Co., Ltd., Shanghai, China) following the manufacturer′s instructions. Then, the RNA was subjected to reverse transcription using the Revert Aid First Strand cDNA Synthesis Kit (Thermo Scientific, United States). RT–qPCR was conducted using SYBR Green II Premix (Takara) in a three‐step amplification procedure according to the manufacturer′s protocol with an ABI 7500 detection system. The reaction conditions were as follows: 95°C for 10 min, 95°C for 10 s, and 40 cycles at 60°C for 30 s. The primers used were designed and synthesized by Sangon Biotech Co., Ltd. (Shanghai, China) and are provided in Table S2. The expression levels of the samples were evaluated according to the cycle threshold (CT) value. The relative expression of CDHR2 in each group, namely, the WT, MT, and NC groups, was calculated using the 2^−*Δ* *Δ*CT^ method.

### 2.7. Western Blotting Analysis

Total protein was extracted from transfected HEK293T cells and quantified using a BCA kit. Protein lysates were separated by sodium dodecyl sulfate–polyacrylamide gel electrophoresis (10%) and transferred onto polyvinylidene fluoride (PVDF) membranes. The membranes were then blocked with 5% skim milk in Tris‐buffered saline containing Tween 20 (TBST) (0.05%) for 2 h at room temperature, followed by overnight incubation with primary antibodies (CDHR2, 1:500, and ATP1A1, 1:2000) at 4°C. The membrane was subsequently washed three times with TBST and then incubated with a secondary antibody (1:5000) for 2 h at ambient temperature. After another wash with TBST, the enhanced chemiluminescence working solution was added to the PVDF membranes for protein blot scanning. The blots were visualized using fluorescence imaging and subsequently scanned for analysis. ATP1A1 and GAPDH were employed as internal controls. The relative protein levels of CDHR2 were normalized to ATP1A1, whereas those of YAP, p‐YAP, TAZ, and p‐TAZ were normalized to GAPDH based on gray intensity analysis.

### 2.8. Immunofluorescence Staining

Following transient transfection for 24 h, the HEK293T cells were segregated into two cohorts: CDHR2‐WT and CDHR2‐MT. The cells were subsequently cultured in a confocal dish for 48 h prior to fixation with 4% paraformaldehyde for 20 min. After being washed with PBS, the cells were incubated with 10% goat serum at 37°C for 30 min to block nonspecific antigens. Staining was performed using primary antibodies against Flag and fluorescent‐labeled human antirabbit secondary antibodies at a dilution of 1:200 (Proteintech Group, Inc.). The nuclei were counterstained with 4 ^′^,6‐diamidino‐2‐phenylindole (DAPI) obtained from Solarbio Technology Co. Ltd. (Beijing, China). The cells were subsequently visualized using a Nikon ECLIPSE Ti confocal microscope, and the resulting images were captured and analyzed using the NIS‐Elements Viewer software (Nikon, Japan).

### 2.9. Statistical Analysis

All the experiments were repeated at least three times. Statistical analysis and graphing were performed using GraphPad Prism software (Version 8.0, GraphPad Software Inc., California, United States). The expression levels of CDHR2 mRNA and protein were presented as the means ± SEs. A significance level of *p* < 0.05 was considered statistically significant ( ^∗^ *p* < 0.05 and  ^∗∗^ *p* < 0.01). Two‐group comparisons were performed using *t*‐tests, and one‐way analysis of variance (ANOVA) was used to compare three or more groups. Additionally, nonparametric tests were employed for data with a nonnormal distribution.

## 3. Results

### 3.1. Clinical Features of the OIT Family

The proband of Pedigree 1 was a 22‐year‐old Chinese girl (II‐3) who was diagnosed at the Fourth Hospital of Hebei Medical University. The second patient in this family was her younger sister (II‐4), who was diagnosed at age 38 at the First Hospital of Hebei Medical University. The other members of Pedigree 1 were healthy, including the parents (I‐1 and I‐2), two elder sisters of the proband, and four offspring of the proband′s generation. The patients in Pedigree 2 were the proband (III‐1), her mother (II‐1), and her grandmother (I‐1). The other individuals (II‐2 and III‐2) were healthy. However, there was a half‐sister relationship between the second‐generation individuals due to their mother′s remarriage. All patients presented typical pathological characteristics of OIT, and their pathological diagnoses were described in our previous paper.

### 3.2. Genetic Analysis

WES revealed that all affected women in the first OIT family carried a nonsense mutation at position 2233 (c.2233C > T) in Exon 19 of the *CDHR2* gene, which was confirmed via Sanger sequencing. This variant was also detected in the patients′ mother (I‐2) but was absent in their father (I‐1) and other immediate family members. It was not detected in the individuals in Pedigree 2. Our study of Pedigree 1 indicated that *CDHR2* c.2233C > T was of maternal origin and *BMP15* c.262C > T was of paternal origin. The two pedigrees and part of the sequencing results at the mutation site are shown in Figure 1. Cosegregation analysis of the mutations and phenotypes according to the 2019 guidelines indicated that the *Z* value was 0.85. Therefore, the variant cosegregated with the phenotype in this family (*Z* ≥ 0.6). The variant was reported to have an extremely low allele frequency (6 × 10^−5^~1.08 × 10^−4^) in several population databases, such as ExAC. Records of this mutation were found only in the dbSNP database (https://www.ncbi.nlm.nih.gov/snp/rs200848414/#hgvs_tab) and the COSMIC database. The clinical significance of this mutation has not yet been reported in the ClinVar database (https://www.ncbi.nlm.nih.gov/clinvar/?term=rs200848414). MutationTaster and CADD results showed that this mutation was deleterious and harmful (the CADD score for the variant was 37, and a score ≥ 20 indicates a harmful effect).

#### 3.2.1. *CDHR2* c.2233C > T Variant Affects the mRNA and Protein Levels of CDHR2

**Figure 2:**
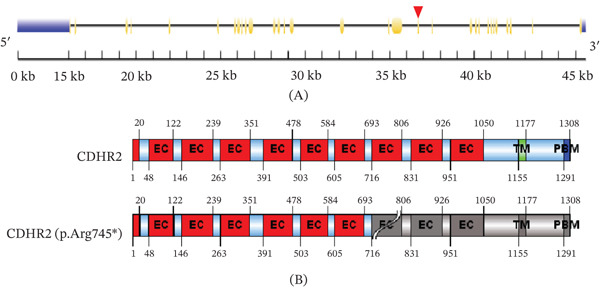
Diagram of the human *CDHR2* gene, protein, and mutant variants. (A) *CDHR2* gene structure. The purple and yellow boxes indicate the noncoding domains and exons, respectively. The mutation site is indicated by the red triangle. (B) The primary sequence of the CDHR2 protein and its variant product (p.R745X). The red and green boxes represent extracellular cadherin (EC) repeats and transmembrane (TM) domains of the protein, respectively. PBM indicates the PDZ‐binding motif of CDHR2. A double slash represents a nonsense terminus.

**Figure 3:**
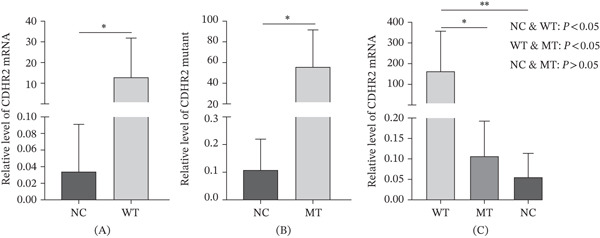
Transfection efficiency and CDHR2 mRNA levels were compared among different groups of HEK293T cells transfected with various plasmids. (A, B) Transfection efficiency. (C) CDHR2 mRNA levels in the various cell groups. NC: HEK293T cells transfected with the GV657 vector; WT: HEK293T cells overexpressing the CDHR2 plasmid; MT: HEK293T cells overexpressing the CDHR2 mutant plasmid.

**Figure 4:**
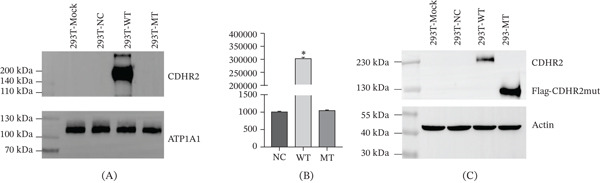
CDHR2 protein levels in the different groups of HEK293T cells transfected with various plasmids. (A) The Western blot results of the CDHR2 polyclonal antibody. (B) The gray values among different groups are displayed. The group identifiers are located at the bottom of the graph. NC: HEK293T cells transfected with the GV657 vector; WT: HEK293T cells overexpressing the CDHR2 plasmid; MT: HEK293T cells overexpressing the CDHR2 mutant plasmid. (C) Immunoblotting analysis of CDHR2 mutant expression in 293T cells with Flag antibody.

*CDHR2* c.2233C > T introduces an early termination codon through substitution of arginine (R) at position 745 of CDHR2 (p.R745X). This site is located in the seventh extracellular cadherin (EC) repeat of CDHR2. A schematic representation of the gene and protein structures was drawn using IBS online (Figure 2). *CDHR2* c.2233C > T can alter the mRNA and protein levels of CDHR2 in vitro. The results revealed a significant reduction (*p* < 0.05) in CDHR2 mRNA and protein expression in CDHR2‐MT HEK293T cells compared with that in WT cells (Figures 3A,B and 4A,B). These findings suggested that the c.2233C > T mutation could lead to a substantial reduction in CDHR2 levels. Flag‐tagged WT and MT CDHR2 plasmids were transfected into 293T cells, and protein expression was examined via immunoblotting with an anti‐Flag antibody. Immunoblotting results showed that the MT CDHR2 protein failed to yield a band corresponding to the predicted full‐length molecular weight. Instead, it produced an aberrant protein product with reduced molecular mass (Figure 4C).

#### 3.2.2. c.2233C > T Variant Alters Subcellular Localization of the Protein

**Figure 5:**
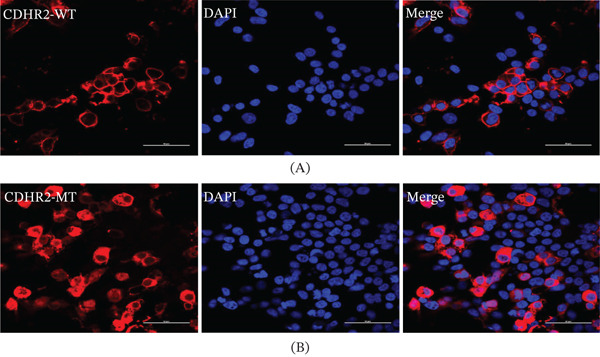
CDHR2 p.R745X altered cellular localization. DAPI was used for nuclear staining (blue); the red fluorescence on the left originated from a Flag combined with an immunofluorescence antibody, while the merged images are displayed on the right. (A) CDHR2‐WT is located on the cell membrane. (B) CDHR2‐MT is located predominantly in the cytoplasm.

To explore the changes in the intracellular distribution of CDHR2 p.R745X compared with that of CDHR2, cellular immunofluorescence technology was used. The results revealed that the CDHR2‐WT protein localized to the cell membrane, which was consistent with the protein localization characteristics of transmembrane (TM) proteins (Figure 5A). However, CDHR2 p.R745X, the CDHR2‐MT protein, from the MT plasmid was located mainly in the cytoplasm, losing the typical localization of a TM protein (Figure 5B).

## 4. Discussion

The majority of tumors result from the combined effects of environmental and genetic factors and are predominantly sporadic in nature. Hereditary tumors account for approximately 5%–10% of all tumors and are caused by pathogenic germline mutations in susceptibility genes. These tumors often exhibit familial clustering and early onset. In our previous study, we identified the germline mutation *BMP15* c.262C > T as a potential susceptibility gene for hereditary OIT [9]. In this study, using comparative genetic analysis of OIT patients and nonpatients carrying the *BMP15* c.262C > T mutation within the same family, we demonstrated that the germline mutation *CDHR2* c.2233C > T may contribute to OIT in conjunction with the *BMP15* mutation. *CDHR2* c.2233C > T is a truncating mutation. In vitro studies revealed that the presence of this mutation caused a significant reduction in the intracellular expression level of the CDHR2 protein and altered the subcellular localization of the MT product compared to the CDHR2‐WT protein.

CDHR2 (also known as protocadherin‐24, PCDH24), a member of the protocadherin family [12], is a TM protein consisting of 1310 amino acids. The protein comprises a signal peptide, nine tandem EC repeats, a TM region, and an intracellular segment with a PDZ‐binding motif (PBM) [13]. CDHR2 plays a critical role in epithelial cell adhesion [14] and contact inhibition [15] and is considered a potential tumor suppressor [16]. CDHR2 is highly expressed in normal liver, kidney, and colon tissues. In contrast, CDHR2 expression is significantly reduced in cancers of these tissues, suggesting that CDHR2 could be a candidate molecular marker and a potential tumor suppressor for these malignancies [17, 18]. To date, no studies have reported the association between CDHR2 and gynecological tumors.

By retrieving public databases such as dbSNP, OMIM, ClinVar, and HGMD, we found that in the general population, *CDHR2* primarily carries benign polymorphic variants, while truncating mutations and missense mutations are rare. The pathogenicity and functions of such mutations are rarely documented. To date, no clinically validated pathogenic phenotypes, tissue‐specific eQTLs, or robust GWAS association signals for *CDHR2* have been established. In the present study, we revealed that the germline mutation *CDHR2* c.2233C > T may be associated with hereditary OIT. Previously, this mutation site in *CDHR2* was identified in exome sequencing data from prostate cancer and colorectal carcinoma patients and was recorded in both the dbSNP and COSMIC databases [19, 20]. However, the function of this mutation site and its relationship with the biological behavior of cancer remain unestablished. Accordingly, the clinical significance of *CDHR2* c.2233C > T has not yet been reported in the ClinVar database. As a truncating mutation, the T allele causes premature termination of *CDHR2* synthesis at amino acid position 744, resulting in a protein product lacking portions of the EC domain, the TM region, and the PBM domain. Further Western blot detection of Flag‐tagged CDHR2 and its MT revealed an obvious band in the MT group with a lower molecular weight than full‐length CDHR2. This result preliminarily suggested that the *CDHR2* c.2233C > T variant potentially induces truncated protein formation. Accumulating evidence demonstrated that the EC repeat region stabilizes the molecular conformation of CDHR2 in a calcium‐dependent manner and mediates its homologous and heterologous adhesion with CDHR5 [21]. This process facilitates apical intermicrovillar adhesion in intestinal epithelial cells, strengthens epithelial barrier integrity, and restricts cell migration [22]. The TM domain anchors CDHR2 to the plasma membrane and maintains its canonical topological structure, with an extracellular N‐terminus and an intracellular C‐terminus. This structural feature enables CDHR2 to link the intracellular cytoskeleton, mediate signal transduction, and regulate microvillar dynamics and cell polarity. Moreover, the intracellular PBM of CDHR2 directly interacts with the PDZ2 domain of Harmonin (USH1C), bridging the actin cytoskeleton to the membrane at microvillar tips. Beyond structural anchoring, this motif also participates in regulating microvillus development, cell polarity, proliferation, and differentiation. Accordingly, we speculate that the *CDHR2* c.2233C > T variant causes protein truncation, which is highly likely to disrupt the physiological functions of CDHR2. Our cytological experiments demonstrated that the *CDHR2* c.2233C > T mutation not only markedly reduced CDHR2 expression but also resulted in the subcellular localization of the MT protein, which was significantly different from that of CDHR2‐WT. Based on these findings, we propose that *CDHR2* c.2233C > T is a pathogenic mutation. According to the ACMG classification standards and guidelines for genetic variants, *CDHR2* c.2233C > T was classified as a pathogenic mutation [23].

Although the *CDHR2* c.2233C > T mutation has been classified as pathogenic, we propose that this variant may act as an enhancer gene or synergistic gene of *BMP15* in the pathogenesis of hereditary OIT. This hypothesis is based on the following observations: (1) In Pedigree 1, the *CDHR2* c.2233C > T mutation was maternally inherited by two patients, yet the mother carrying this mutation was healthy. (2) In Pedigree 2, the mutation was not detected in the mother–daughter patient pair. How does CDHR2 synergize with BMP15 in the development of OIT?

Accumulating clinical and experimental evidence indicates that ovarian teratoma (OT) originates from aberrant parthenogenetic activation of oocytes, followed by dysregulated embryonic development and disordered tissue differentiation [24–26]. Our previous in vivo studies demonstrated that the *BMP15 c.262C > T* variant facilitates excessive oocyte parthenogenesis through activation of the BMP15/GDF9‐SMAD‐MAPK signaling cascade, which serves as an initiating event for OIT tumorigenesis [9]. Nevertheless, the molecular mechanism by which parthenogenetically activated oocytes lose developmental control and ultimately form disordered three‐germ‐layer structures characteristic of OIT remains poorly understood.

Previous mammalian embryology studies have illustrated that maternal depletion of YAP/TAZ leads to developmental arrest of fertilized eggs and blastomeres, which fail to reach the blastocyst stage [27]. Specifically, ablation of maternal YAP/TAZ at the four‐cell and eight‐cell embryonic stages induces premature SOX2 expression prior to the fourth cleavage, which subsequently downregulates caudal‐type homeobox 2 (CDX2). This molecular disturbance disrupts the balanced differentiation of inner cell mass and trophectoderm lineages, thereby causing severe embryonic developmental defects [28]. Collectively, these findings suggest that HIPPO pathway–dependent differentiation homeostasis is critically involved in modulating germ cell developmental fate and may contribute to OIT pathogenesis.

**Figure 6:**
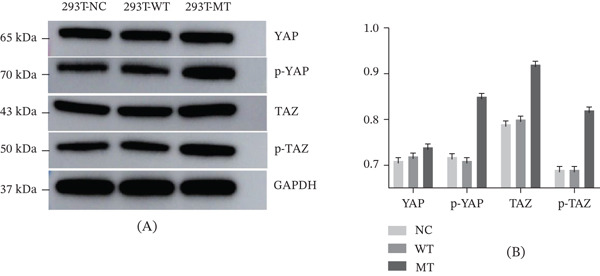
Expression levels of HIPPO pathway–related proteins in each group. (A) Immunoblotting analysis demonstrated that the protein abundances of p‐YAP and p‐TAZ were markedly increased in 293T cells of the mutant group relative to the control and wild‐type groups. (B) Quantitative grayscale analysis of YAP, p‐YAP, TAZ, and p‐TAZ protein bands in each experimental group.

CDHR2 is essential for microvillus morphogenesis, cellular polarity maintenance, and the fine regulation of cell proliferation and differentiation [14, 29]. Given the fundamental biological functions of CDHR2, we hypothesized that pathogenic CDHR2 variants may disrupt normal cellular differentiation by altering HIPPO signaling activity, thereby contributing to the occurrence of hereditary OIT in this pedigree. To test this hypothesis, we examined the expression patterns of core HIPPO pathway components in three groups of cells (Figure 6). The results showed markedly increased phosphorylation levels of YAP and TAZ in MT cells, which trapped YAP/TAZ in the cytoplasm for degradation and reduced their nuclear translocation. This aberrant HIPPO activation perturbs the normal differentiation program of ovarian germ cells, induces developmental disorder at the early tumorigenic stage, and ultimately facilitates abnormal tissue differentiation and OIT occurrence.

While no CDHR2 mutations were detected in affected individuals from the second OIT pedigree, two germline CDHR2 variants (rs2291442, p.T1128M, and rs1707834, p.L1164M) were uncovered in our ongoing WES analysis of a mature OT pedigree harboring *BMP15* polymorphic loci. Structural functional annotation confirmed that both variants reside within the key functional domains of the CDHR2 protein. The recurrent identification of CDHR2 genetic alterations in distinct OT subtypes demonstrates that these variants are not random genetic fluctuations but possess genuine biological significance. These findings further validate that functional deficiency of CDHR2 is closely implicated in the abnormal differentiation characteristic of teratoma pathogenesis.

Interestingly, a study by Naser et al. demonstrated that mice carrying double mutations in *MC4R* and *DND1* presented a significantly greater incidence of OTs [30]. Their research indicated that the missense mutation in *MC4R* promoted teratoma formation in the presence of germ cell abnormalities caused by the *DND1* mutation. These findings suggest that the pathogenesis of hereditary OIT may involve the interplay of multiple genes. The *CDHR2* c.2233C > T mutation was not detected in the affected individuals of the second family. Unlike in the first family, the *BMP15* c.262C > T mutation was maternally inherited in the second family. In the second generation of the second family, a half‐sister with the same mother but a different father did not carry the germline *BMP15* mutation, adding complexity to the identification of other differential gene mutations within this family. This finding suggests that, in the pathogenesis of hereditary OIT, while *BMP15* acts as the primary effector gene, enhancer or synergistic genes may vary between families. This result highlights the heterogeneity of genetic foundations for the same disease and the possibility that complex diseases may involve multiple genes. Given the findings of the present study, our group will further perform mass spectrometry profiling and systematic functional validation of MT CDHR2 proteins to characterize the molecular features and biological functions of altered CDHR2 products. Meanwhile, expanded recruitment of clinical samples is required to further elucidate the etiological mechanisms underlying hereditary OIT, which will help refine future preventive and therapeutic strategies for this disease.

## 5. Conclusion

Our study suggests that the occurrence of hereditary OIT may result from genetic mutations in multiple genes. *BMP15* is likely the primary effector gene, whereas *CDHR2* may represent one of several enhancer or synergistic genes. Different pedigrees may carry distinct modifier genes, reflecting obvious genetic heterogeneity. This study provides new genetic and functional evidence for understanding the pathogenesis of familial OIT. The identification of additional susceptibility or synergistic genes will require further investigation with more familial cases.

## Author Contributions

Shan Kang and Yan Li conceived and designed the study. Yakun Liu provided clinical data. Yuntao Hao generated experimental data and analyzed all data. Hongwei Fan supervised the analyses. Yuntao Hao and Xi Kang wrote the initial draft of the article. Yuntao Hao and Xi Kang contributed equally to this work and should be considered co‐first authors.

## Funding

This study was funded by the Nature Science Foundation of Hebei Province, Grant No. H2020206535, and the Innovative Funding Project for Graduate Students, Grant No. CXZZBS2022083.

## Disclosure

All authors reviewed the article and approved its final version.

## Ethics Statement

The studies involving human participants were reviewed and approved by the Fourth Hospital of Hebei Medical University Research Ethics Committee (No. IACUC‐4th HosHebmu). Written informed consent was obtained from the individuals.

## Conflicts of Interest

The authors declare no conflicts of interest.

## Supporting information


**Supporting Information** Additional supporting information can be found online in the Supporting Information section. The sequence of primers used in this study are shown in the Supporting Information tables.

## Data Availability

Data available on request from the authors.
