# Supplementary material for: CDHR2 c.2233C > T Is Involved in Human Familial Ovarian Immature Teratoma With BMP15 c.262C > T
Source: Hum Mutat. 2026 Jun 19;2026:8441244. doi: 10.1155/humu/8441244 (PMC13280461; doi:10.1155/humu/8441244)
Supplement: Supplementary file 1 — Supporting Information Additional supporting information can be found online in the Supporting Information section. The sequence of primers used in this study are shown in the Supporting Information tables. [file HUMU-2026-8441244-s001.docx]

**Supplementary Table 1:**

Primers of CDHR2 used in Sanger sequencing for genotype validation in OIT pedigrees.

| **Primer** | **Sequence** |
| --- | --- |
| I | F: GACACTGCGGCTGGGACCCAG |
| II | R: GTTCACGTCTTTCACATTCACG |

**Supplementary Table 2:**

Primers used in Reverse transcription and RT‒qPCR in vivo experiments

| **Primer** | **Sequence（5’-3’）** |
| --- | --- |
| CDHR2-WT  NM_017675 | F:GTGTCATCATAGGATTGGGAGT |
|  | R:GTCTTTGTTGGGGAGGTTCA |
| CDHR2-MT  NM_017675(1-744aa) | F:CAGCTATGGCTGTCCTGCTT |
|  | R:GGTCATTGTCCTGGTCTTCC |
| GAPDH | F:ACCACAGTCCATGCCATCAC  R:TCCACCACCCTGTTGCT GTA |
